# The Psychosocial Experience of Adolescents with Haematological Malignancies in Jordan: An Interpretive Phenomenological Analysis Study

**DOI:** 10.1155/2014/274036

**Published:** 2014-01-05

**Authors:** Omar Al Omari, Dianne Wynaden

**Affiliations:** ^1^Children and Young People's Mental Health, Faculty of Nursing, Jerash University, P.O. Box 311, Jerash 26150, Jordan; ^2^School of Nursing and Midwifery, Curtin Health Innovation, Research Institute, Curtin University, GPO Box U 1987, Perth, WA 6845, Australia

## Abstract

The qualitative research method of interpretive phenomenological analysis was used to explore the lived experience of 14 Jordanian adolescents with haematological malignancies. They were admitted to two hospitals in Jordan and were interviewed for this study twice during the first six months after receiving their diagnosis. The results of this study revealed three themes: (1) Being in hospital, (2) The changing self, and (3) Fearing the unknown. When the participants were hospitalised due to their illness they were removed from their families and friends and prevented from engaging in their normal daily routine. Participants also reported receiving limited emotional and psychological support from health team members during hospitalisation. From the onset of cancer treatments, the bio-psychosocial side effects of the chemotherapy became one of the most distressing factors for participants affecting all aspects of their life and generated uncertainty about their future. The findings add to existing understanding of the lived experiences of cancer patients and in particular Jordanian adolescents. They provide a valuable insight for clinicians into improvements in service delivery to this group of patients.

## 1. Introduction

Jordan is located in the heart of the Middle East and has a population of approximately six million people with young people under the age of 18 years of age making up to 37% of the total population. Jordan has one of the most advanced health care services in the Arab region, and many people come from other Arab countries to receive high level tertiary care for illness such as heart disease and cancer [1]. In Jordan, a significant number of adolescents live with cancer [2]. In 2007, the rate of cancer for patients aged between 10 and 19 years was 132 new cases per 100,000 populations and the survival rate of these adolescents is poor when compared to their counterparts in other ages groups [3, 4].

When an adolescent is diagnosed with HMs, delays in treatment decrease the possibility of remission, worsen the condition, and increase the possibility of complications and death. However, treatment exposes the adolescent to a range of different physical, psychological, social, and/or spiritual experiences [5]. Their lives and that of their family changed forever from the time the diagnosis is made [6, 7]. The adolescent endures long periods of hospitalisation and invasive treatments [5, 7] as health care providers seek improved outcomes for their patient. The adverse effects of this experience for the adolescent are alterations in their body image, low levels of self-esteems, decreased social relationships [7], poor peer acceptance, experiences of stigma, risks for depression, and abnormal levels of stress [8, 9]. During hospitalisation, they may also witness the death of other adolescents who had similar illnesses to themselves [10]. HMs also impact the social world of the adolescent; for example, many have decreased rates of school attendance [11, 12] and their illness impacts their educational outcomes and social development [13]. Many people in Jordan also believe cancer is contagious and a life threatening disease and stigma is a major issue [14] and as a result the affected person is excluded from society at many levels. Chemotherapy also has many adverse psychosocial effects on the patient, for example, on their perceived body image [8, 15, 16]. Chemotherapy side effects change the adolescent's appearance and these changes may have a profound effect on their level of wellbeing. Wallace et al. [17] found that this was one of the most stressful experiences that adolescents face living with cancer as due to their appearance they were often excluded or stigmatised by their community [18]. Compounding this experience is the adolescent's developmental stage. It is during this time that they experience rapid physical and emotional changes associated with puberty and having cancer adds a stressor to their already challenging and changing world.

While many of the adverse psychosocial effects that adolescents with HMs experience have been identified in the global literature, there is still a need to examine their impact longitudinally across the illness journey. It is also important to determine the impact of the unique experiences of Jordanian adolescents as there is currently a dearth of studies in this area and hence the aim of this qualitative study was to explore, understand, and describe the lived experiences of Jordanian adolescents with HMs.

## 2. Materials and Methods

The qualitative research method of interpretive phenomenological analysis (IPA) methodology was used to describe the experiences of Jordanian adolescents living with HMs. This methodology is best suited and valuable when little information is known about the phenomenon being studied [19] as it allows complex descriptions of the experiences to be described and documented. As such, greater insights are provided into the adolescent's illness journey and the resultant behaviours and actions displayed by them [20].

### 2.1. Sample/Participants

Ethical approval to conduct the study was obtained from one university and two hospitals ethics committees. Written consent was obtained from both adolescents and their parents prior to starting the interview.

Participants were included in the study if they agreed to participate and (1) had been diagnosed with a leukaemia, Hodgkin's lymphoma, or non-Hodgkin's lymphoma; (2) had received the diagnosis within three months of the data collection period; (3) were aware of their diagnosis before being interviewed; (4) aged between 13 and 17 years; (5) were from Jordan; (6) did not have a mental or physical condition that affected their ability to understand the researcher's questions or to express their experiences of living with HMs; and (7) understood the reason for the interview and together with their parents gave informed consent. Fourteen participants were interviewed on two different occasions: (1) within three months of their diagnosis of HM and (2) within three months after the initial interview. This sample size is considered adequate to achieve the objectives of the study when using IPA methodology [21]. Data were collected using in-depth semistructured interviews [22, 23] which were audiotaped, using a USB recorder. All interviews were conducted in a private, mutually agreed upon location with adequate lighting and acoustics distance at hospitals.

### 2.2. Data Analysis

Data were transcribed verbatim by the researcher soon after each of the interviews was conducted. These interviews were then reviewed for accuracy, by the researcher, against the original data recording. The data were managed using ©QSR NVivo8 software. This software facilitated management and coding of the transcribed data easing the extrapolation, emergence, and identification of themes. Data analysis was conducted using the method of IPA described by Smith et al. [21] to identify the emergence of themes. Using qualitative steps to ensure the trustworthiness of data, two researchers independently conducted the analysis on the data to set the emergence of themes. They searched for a connection across the emergent subthemes and collapsed them into major themes.

### 2.3. Rigour

The researchers rereviewed the data to confirm that the interpretation and description were appropriate and progressed to meet the standards of qualitative analysis [24]. The two researchers then reexamined at the data together and differences between the researchers themes identification were discussed until acceding was achieved. While this process consumed a substantial time it augmented the trustworthiness of data [22].

## 3. Results

Demographic data on participants is detailed in Table 1.

Three major themes emerged from the data: being in hospital, the changing self, and fearing the unknown. These themes were identified by all participants and were central to the lived experience of their journey with cancer.

### 3.1. Being in Hospital

Being in hospital was the central experience for participants during the first six months following their diagnosis. When hospitalised, participants were subjected to a number of strict rules that placed boundaries on their daily routine and lives. For example, Participant three felt constrained by the four walls of the ward. She compared her life prior to diagnosis as one of a different and satisfying routine of going to school, studying, and playing with friends. The prolonged period of hospitalisation altered all of the things she had previously taken for granted in life. Due to illness and hospitalisation, there is a new limit placed on her that she had not previously experienced:

*I like my school and considered it as my second home … I used to spend seven to eight hours every day at school. In school, I played some games with my friends like hide [and] seek and the circle game. I used to play with them for hours and I used to study with my friends every day. Now I cannot do any of these things and I constrained between four walls … I am very stress in this place and we do not have facilities to communicate and study with our friends and there is no place to play. (Participant 3, Interview 1)*

When participants were hospitalised they were subjected to new hospital routines and they were unable to continue their regular activities which increased their level of stress. Participant two spoke of how this made him feel sick inside: “I do not like hospital … I do not like the routine [in the hospital]. The most annoying things are the doctors … Doctors came and woke me up for weight measurements in the morning, after that they woke up me for my temperature then for medical rounds or in the evening for another temperature reading” (Participant 2, Interview 1).

Participants were also unable to see all their family and friends as a result of their hospitalisation and this was isolating for them as Participant two explained:

*… Here [in the hospital] I feel bored because I'm isolated in the room. I cannot get out; there is nobody to visit me [friends]. They [doctors and nurses] did not allow my friends to visit me. A person should be able to go out, walk and have fun. (Participant 2, Interview 1). Hospitalisation deprived the adolescents with HMs from being with their family members and friends, which increase the illness severity.*

The hospital routine and the new restrictions that participants were forced to adhere to compounded their illness experience and increased their suffering. One participant described the impact of these restrictions on her wellbeing: “I need everyone to visit me,… they will relieve my suffering” (Participant 10, Interview 2).

Participants felt deprived of physical activity and of the experiences of being with their friends. They had to take on new roles and routines that were alien and often conflicted with their previous life experiences. Consequently, due to their limited freedom and lack of autonomy within the hospital environment they were deprived of studying, socialising, and playing with their friends, which further increases their level of anxiety. Within this social context adolescents started to experience the changing self.

### 3.2. The Changing Self

As participants' illness progressed it led them to have negative feelings and experiences about themselves. These negative feelings and experiences were further exacerbated by their developmental stage, which is a period that is heavily focused on identity, peer group interactions, and physical and emotional achievements. The illness brought about changes to their physical appearance which particularly impacted strongly on their gender identity. The side effects of their chemotherapy resulted in dramatic changes in their physical appearance and Participant four provided an example of her assessment of this believing she was not beautiful anymore. Before commencing chemotherapy, she was very proud of her long hair and she did not often wear a Hijab (head scarf). After chemotherapy she wore the Hijab all the time to cover her baldness and her distress of her changing physical appearance. She also described herself as previously having a good sense of humour which she believed she had now lost after her diagnosis of HM:

*I used to not wear a Hijab before my sickness but now I wear it all the time. Sometimes I was wearing a pyjama, blouse and jeans while making my rounds to see my friends. … My hair was long and I was pretty, not like now. I do not have any hair and my shape has changed. I was beautiful [before I became ill]. … Compared to what I looked like before I am not that beautiful. (Participant 4, Interview 2)*

The changes in participants' physical appearance affected their level of psychological wellbeing. They started to complain of low self-esteem and Participant 11 expressed concerns about her femininity. She reflected on how she now looked like a boy due to her hair loss and believed that her new self-image would frighten her friends away and they now avoided her. When Participant 11 was talking about the things that were the hardest for her during as a result of her illness she stated:

*The hair loss [pause] I am a girl and I liked my hair. Without hair, I feel that I am like a boy and I do not know how my friends will respond when they see me like this [without hair]. They [friends] will not like it [my new look]. (Participant 11, Interview 2)*

At the time of being interviewed, participants were not receiving education to assist them to manage the impact of the physical changes on their level of wellbeing. They felt anxious, vulnerable, and shameful regarding what was happening to their body. They began to stigmatise themselves as they believed that other people would reject them if they knew they had cancer. Participant six tried to gauge what kind of response he might expect from his school friends if he told them about his diagnosis by revealing the information in an anonymous internet chat room. His resulting experience was catastrophic for him and he experienced stigma from people he had never personally met. They called him the “cancer boy.” After this experience, he kept his diagnosis secret staying silent about his illness for fear of further rejection:

*I told them [people in the chat room] yes [I have cancer], so they would leave me and go far away from me. I don't know why, and they called me “cancer boy”. In particular, a girl who I knew before [I got sick] she disturbed me with this title. … I went to her house [on the internet] and shouting that all people have cancer, not just me. She said to me “Don't talk to me”. At that time I thought very seriously about leaving everyone [to isolate myself]. (Participant 6, Interview 2)*

Participant seven also feared rejection and this was based on previous experience. She made a comparison between herself and a friend who had a bad experience when she told people she had cancer. Participant seven did not want to inform anyone about her illness because she knew that she could lose friends too if she disclosed to them. At the time of the second interview, Participant seven had communicated with her friends via the internet but she was still not willing to tell them that she had an HM:

*I expect that the students will ask me why I have been absent from school. This is an expectation, so I don't care about the answer. [Pause] I will answer that I was sick without telling them my real diagnosis. I don't want to tell them; even on the internet when I connect with my friends, they ask me “What's wrong with you?” I just tell them that I'm tired. … We have a girl in school who had a brother who was sick with this disease. Just imagine, when her brother had this disease, the girls kept away from her, they were scared of the infection! You feel that people will keep away from you, so why should I put myself in the same situation? (Participant 7, Interview 2)*

Having an HM and undergoing chemotherapy had a deep effect on all participants as they moved through the experience of a changing self. They could no longer trust their bodies or their physical capabilities and they were scared of losing their gender identity. They were frightened to disclose information regarding their illness because they may be rejected and stigmatised by their friends. They felt alone and in fear of the unknown.

### 3.3. Fearing the Unknown

Participants were not prepared for the side effects of chemotherapy or the invasive diagnostic tests or treatments that were conducted on them. They also began to come to the realisation that they may die sooner than they had previously expected. Misinformation from other patients about tests and treatment they were scheduled to have further exacerbated their anxiety and fear. Bone marrow aspirations which were frightening and painful were particularly stressful as participants knew they could experience complications as a result of the procedure. Their fear of the procedure was made worse by the lack of accurate information and education provided to them from health professionals. For Participant one, being presented with the news that he had to undergo a bone marrow aspiration procedure filled him with fear. He had received no information from health professionals prior to the procedure and as a result he became disabled by the fear and the doctors were unable to complete the procedure. However, after talking with other patients he agreed to undergo the procedure:

*… Then they [the doctor] told me that they wanted to take a bone marrow biopsy, but I told them that I would not let them take it, because I knew nothing about it and I was afraid. [Pause] I fear pain and fear of becoming paralysed, but at the end some other patients convinced me to do it. (Participant 1, Interview 1)*

Participant nine also experienced fear of the unknown due to a lack of information and preparation from the health team members: “No, no one told me that the bone marrow aspiration would be painful, but I became afraid of the bone marrow biopsies until the last one, when they told me that they would anesthetise me. … They anaesthetised me, so I felt no pain” (Participant 9, Interview 1). As a result of this experience, Participant nine became sensitive and fearful of all future procedures or surgery. He also believed that the bone marrow aspiration was the first of a series of more invasive procedures: “… it is because I think that a serious thing has happened [due to my bone marrow aspiration], and now I'm afraid that they will perform another operation on me.” (Participant 9, Interview 1).

The lack of knowledge, education, and support leads participants to reflect on their own mortality. Participant eight saw the people around him dying and he heard unpleasant stories about the outcomes for people who have cancer. He used the word “dominant” to describe the impact and size of his thoughts of his potential death on his life as it became an important, dominant, and consuming focus of his life:

*… Yes, everyone thinks about death. … One in my circumstances and my situation thinks about death. After I got cancer, in the early stages, I did not know what the nature of my sickness was and no one tell me, but when I saw people and they began to tell me unpleasant stories about cancer, and I saw people die, the idea of death became dominant in my life, cancer is a killer. (Participant 8, Interview 2)*

Participant 11 faced uncertainty about her future; she had a fear of being alone, and this became pronounced as she became more unwell. Death became a complex reality for her and she began to imagine what her life would be like when she died:

*… I'm scared of not seeing my family, and staying in the grave alone. I thought about death when I was very tired, when I was admitted to the ICU. I even told my mum, if I died, to bury me near the house because I'm afraid to be alone. (Participant 11, Interview 2)*

Participants began to understand the complex nature of their diagnosis of HM. They reflected on the changes that this diagnosis made to their lives. They had lost their normal life routine and many of their friends. They were also faced with the reality of their own death. Stigma was a major issue for participants and greatly impacted their ability to cope with their diagnosis of HM. Due to stigma they lost their friends and they experienced isolation from their previous routine and life before being diagnosed with HM.

## 4. Discussion

### 4.1. The Negative Impacts of Hospitalisation

While hospitalised participants were prevented from engaging in their normal daily routine and doing many of the activities that they used to do, for example, catch-up with their family and friends and going to school. These effects of hospitalisation on adolescents have also consistently been reported in the literature [10, 12, 25, 26]. Adolescents both in this current study as well as in the existing literature highlighted the importance of and their need for support during their illness journey from their families and peers [18, 26–28]. Previous studies also signify the importance of cancer patients supporting each others as these supports allow them to discuss sensitive issues [11, 26, 29]. In general, support provided patients with stability in their lives and protected them from unpleasant experiences [8, 18, 26]. Several other researchers have found that being away from these important support structures caused major difficulties for adolescents with cancer to cope while being hospitalised [30–32]. Therefore, these important psychosocial support factors need to be incorporated into oncology care to keep adolescents in touch with their families and friends. This can be achieved through more family centred care and family friendly hospital environments where visiting hours are flexible and provide families with strategies to provide a high level of support to their child.

The International Society of Paediatric Oncology (SIOP) working committee on psychosocial issues in paediatric oncology has stressed the significance of a therapeutic alliance between health team members and the patient's family in order to reduce participants negative experiences [33]. There is a need to reevaluate current approaches to care in Jordanian hospitals and adopt new policies and practices that ensure the delivery of a family centred care is implemented for the wellbeing of the affected adolescent during their illness journey.

### 4.2. The Biopsychosocial Adverse Effects of Cancer

Many of the participants in this study experienced adverse biopsychosocial effects as a result of their chemotherapy treatments. Other studies have also identified that losing hair (alopecia) from the body was the most devastating effect of chemotherapy and hair loss greatly impacted the patient's body image [17, 34, 35]. It is well established that negative body image can have a detrimental effect on the mental wellbeing of the adolescents [30, 36, 37]. As a result of changes in their body image, participants reported low levels of self-esteem and descriptions of feelings that are consistent with those expressed by people with depression and high levels of stress. These findings are consistent with other research [16–18].

The alteration in body image due to alopecia was further exacerbated because it led to an additional threat to their developing gender identity. For women particularly, hair is a symbol of their identity, beauty, and femininity [38] and research has shown that female adolescents with cancer are more concerned about their appearance than males [35]. However, in this study, males and females were equally concerned about their hair loss and its impact on their body image and gender identity. This is seen particularly important as in Jordanian society hair is linked to gender identity and the associated values and boundaries that separate male and female roles.

Participants were also concerned about dying, pain, and invasive procedures and treatments further compounded this fear and concern. This relationship between pain and the possibility of death has also been previously described in the literature [39–41]. The negative impacts of cancer and the related psychosocial changes in participants exposed them to the risk of developing comorbidities which may prolong their length of stay in hospital, negatively impact their treatment journey, and test their resilience and coping skills during this critical time in their lives [42–45]. Another reason that could place participants at increased risk of developing disorders such as depression is social stigma that participants in this study experienced. Stigma directed to cancer patients has been previously identified by other researchers [11, 18, 29, 39, 46, 47]. In previous studies peers of adolescents patients teased them, avoided them, and asked them questions that increased their level of stress [18]. They also have experienced similar stigmatisation from their teachers [11, 29, 35]. Social stigma prevented participants from seeking support from their friends and relatives. Support remains a critical issue for cancer patients as it allows the patient to discuss sensitive issues [8, 26, 41, 48]. There is a need to reduce stigma through resolving the myths that many people hold regarding the causes of cancer. The use of mass media to provide this education would decrease the level of stigma experienced by cancer patients and increase their level of support.

### 4.3. Educational Needs

Participants alluded to their educational needs and the lack of education provided to them by health professionals. However, adolescents in other countries actively sought more education about their illness [11, 12, 18, 49]. They wanted practical tips on how to manage their altered body image [11, 18, 49] and more general information about their illness, for example, exercise, nutrition, complementary and alternative health services, infertility information, mental health counselling, and camp or retreat programs for young adults [49]. They also wanted to know how to cope with embarrassing situations that resulted from the physical changes and resulting negative body image they were experiencing [11]. In addition, they reported a need for more information regarding their illness such as the possibility of the illness recurring and the effects of drugs on participants [11]. Patients in current study need also to be provided with this information. Therefore, health team members are required to develop educational plans that address all the adolescents with cancer educational needs.

### 4.4. Recommendations for Future Studies

Additional qualitative, quantitative, longitudinal, prospective research studies are needed to explore the lived experiences of adolescents with HMs to further identify the developmental changes that they experience during their illness journey. There is also a need for follow-up studies that test the impact of adopting the recommendations from this study on patient outcomes. Furthermore, As this study is one of the first to be conducted in Jordan in the area of adolescents with HMs the researchers recommend further investigations arising from the findings of this study to facilitate the delivery of culturally sensitive quality nursing care.

## 5. Conclusion

This study details the lived experiences of Jordanian adolescents with HMs along with the factors that impact these experiences. The findings demonstrate that adolescents with HMs are exposed to a variety of negative psychosocial experiences throughout their illness journey and they were unprepared to face theses negative experiences. These findings emphasise the importance of educating the adolescents, their parents, and health team members about cancer and chemotherapy in general to decrease the magnitude of the negative experiences.

This study has limitations due to homogenous small sample size, which impacts transferability of results to other practice settings. Furthermore, as each of the participants' parents were present during the two data collection periods and their presence may have impacted the data provided. It was compulsory that the parents were present because they were under their fathers' guardianship. Moreover, ethical approval in Jordan required the presence of a psychologist during the interviews to monitor the adolescent's level of wellbeing. However, with these limitations, the researcher, an experienced oncology nurse, was able to establish rapports with the adolescents which allowed for rich and descriptive data to emerge during the interviews.

## Figures and Tables

**Table 1:** Profile of participants.

Item	*N*	%
Participants' age		
13.00	5	36.0
14.00	2	14.0
16.00	3	21.5
17.00	4	28.5
Gender		
Male	9	64.0
Female	5	36.0
Participants' diagnosis		
Hodgkin lymphoma	5	35.0
Non-Hodgkin lymphoma	3	21.0
Leukaemia	6	44.0
Participants work		
Student	13	93.0
Farmer	1	7.0
Type of treatments		
Chemotherapy	14	100
Radiotherapy	0	0
Bone marrow transplant	0	0
Parents' income/year		
Less than 3000 JD	10	71.5
4000–6000 JD	3	21.5
7000–9000 JD	1	7.0
